# Kaempferol in inflammatory bowel disease: regulating intestinal epithelial-fibroblast crosstalk

**DOI:** 10.3389/fphar.2026.1799034

**Published:** 2026-09-22

**Authors:** Haixin Qi, Guiqun Zhou, Yunfei Dong, Xingwei Yu

**Affiliations:** 1 Department of Anorectal Surgery, the First Affiliated Hospital of Zhejiang Chinese Medical University (Zhejiang Provincial Hospital of Chinese Medicine), Hangzhou City, Zhejiang Province, China; 2 Department of Gastroenterology, the Third Affiliated Hospital of Zhejiang Chinese Medical University, Hangzhou City, Zhejiang Province, China; 3 Department of Urology, the First Affiliated Hospital of Zhejiang Chinese Medical University (Zhejiang Provincial Hospital of Chinese Medicine), Hangzhou City, Zhejiang Province, China

**Keywords:** fibroblasts, inflammatory bowel disease, intestinal epithelial cells, kaempferol, tumor necrosis factor α

## Abstract

**Introduction:**

Inflammatory bowel disease (IBD) has an intricate pathophysiology that greatly impacts patients’ quality of life. Wumei Wan, a traditional Chinese medicinal formulation, exhibits specific therapeutic effects on IBD. Kaempferol (KF), an active ingredient in Wumei Wan, may significantly contribute to IBD.

**Methods:**

A 2% DSS-induced chronic colitis mouse model was established, randomly divided into six groups and treated with KF. Relevant indicators of chronic colitis were measured, including body weight, Disease Activity Index (DAI), colon length, histological alterations, as well as indicators of inflammatory factors, fibrosis, intestinal barrier function, hepatic and renal function, and oxidative stress. TNF-α was employed to stimulate colonic epithelial cells in cell studies. After KF intervention, cytotoxicity, transforming growth factor-β1 (TGF-β1), and cell survival were observed to investigate its impact on IBD. The HT29/CCD-18Co medium with TNF-α/TNF-α+KF was co-cultured with CCD-18Co/HT29 cells, and a cell line with heme oxygenase 1 (HMOX1) silencing/overexpression was generated to explore the impact of KF on intestinal epithelial cell-fibroblast crosstalk.

**Results:**

*In vivo*, KF therapy increased body weight, reduced DAI, and improved colon length as well as histopathology. Furthermore, KF reversed the elevated levels of inflammatory cytokines, intestinal barrier function, and oxidative stress markers observed in the model group. *In vitro*, KF reversed TNF-α-induced decrease of intestinal epithelial cells, TGF-β1 downregulation, regulated cell function, and reduced inflammation. KF may suppress TNF-α-induced fibroblast activation, and HMOX1 was crucial for regulating cell crosstalk.

**Discussion:**

Collectively, KF mitigates IBD by modulating intestinal epithelial-fibroblast interaction, suppressing inflammatory responses, reducing intestinal fibrosis, and increasing intestinal mucosal barrier function.

## 1 Introduction

Inflammatory bowel disease (IBD), as a chronic and recurrent intestinal inflammatory disease, seriously affects the quality of life of patients (Singh and Bernstein, 2022). Its pathogenesis is extremely complex, involving multiple aspects such as abnormal immune system, imbalance of intestinal microbiota, and disorder of the interaction between cells in the intestine (Cosín-Roger, 2024). Although various biologics and small-molecule drugs are currently available for IBD treatment, such as anti-TNF-α agents, JAK inhibitors, and IL-23 inhibitors, approximately 30% of patients show no response to initial therapy, and 40% of patients who respond initially experience secondary treatment failure during subsequent therapy (Singh et al., 2018; Zhao et al., 2022). Traditional Chinese medicine compound prescriptions have a long history and extensive practical experience in the treatment of IBD. Wumei Wan, as a classic traditional Chinese medicine formula, contains a variety of herbs such as Smoked Plum, Sichuan Pepper, Manchurian Wildginger, Coptis chinensis Franch, Amur Corktree Bark, Prepared Common Monkshood, Cmnamomi Mmulus, Ginseng, and Chinese Angelica (Wu et al., 2020). A previous studies have shown that Wumei Wan can effectively improve intestinal fibrosis related to chronic colitis by inhibiting the activation of fibroblasts, providing a highly potential strategy for the treatment of IBD (Wu et al., 2020).

Among cellular interactions in the intestinal tract, the crosstalk between intestinal epithelial cells and fibroblasts plays a key role in the occurrence and development of IBD (Rizzo et al., 2023). Under normal circumstances, there exists a delicate regulatory mechanism between intestinal epithelial cells and fibroblasts to maintain the stability of the intestinal internal environment. However, in response to inflammatory stimuli, intestinal epithelial cells secrete various pro-fibrotic factors, such as transforming growth factor -β (TGF-β) (Marincola Smith et al., 2021). These factors can induce activation of intestinal fibroblasts and their extensive proliferation, which promotes the synthesis and deposition of extracellular matrix and leads to the occurrence of intestinal fibrosis (Rieder et al., 2024). Meanwhile, activated fibroblasts also secrete a series of cytokines and chemokines, including interleukin-6 (IL-6) and monocyte chemoattractant protein-1 (MCP-1). These substances can further aggravate the intestinal inflammatory response, form a vicious cycle, and accelerate the progression of IBD (Rieder et al., 2024).

The first-line drug mesalazine effectively alleviates symptoms in patients with mild to moderate IBD (Gordon et al., 2011), but its efficacy is limited in severe cases. Kaempferol (KF) is a common chemical component in Smoked Plum, Manchurian Wildginger and Ginseng, and it has various biological activities, such as antioxidant, anti-inflammatory and antibacterial properties (Periferakis et al., 2022; Chen et al., 2022). In recent years, the potential role of KF in the treatment of intestinal diseases has garnered increasing attention (Chen et al., 2023), and is expected to become an alternative or candidate therapeutic agent for IBD with potential advantages over mesalazine in efficacy and multi-target regulatory properties. Studies have found that KF has a definite therapeutic effect on experimental colitis (Qu et al., 2021). Specifically, in the mouse model of colitis induced by dextran sulfate sodium (DSS), KF exerts a protective effect by regulating the intestinal flora and Toll-like receptor 4 (TLR4)-related signaling pathways (Qu et al., 2021). KF can also play an anti-inflammatory role via maintaining the integrity of the intestinal barrier, protect the tight junction proteins of intestinal epithelial cells (Zonula occludens-1 (ZO-1) and claudin-3), and prevent the impairment of intestinal mucosal barrier function (Wang et al., 2019). Importantly, KF can modulate TGF-β-induced C2C12 myocyte-fibroblast crosstalk, exerting an anti-fibrotic effect (Cuijpers et al., 2025). We hypothesize whether KF can regulate the epithelial-fibroblast interaction.

Furthermore, KF can activate HMOX1 to repress lipid accumulation in macrophages, and the activation of the Nrf2/HMOX1 pathway has been proven to alleviate oxidative stress injury in ulcerative colitis (Li et al., 2013; Wang et al., 2023). Based on the existing studies, we inferred that KF may mitigate IBD via modulating intestinal epithelial-fibroblast crosstalk. This study therefore investigated this regulatory mechanism, aiming to provide novel targets and a theoretical foundation for treating IBD.

## 2 Materials and methods

### 2.1 Animal ethics

In this study, 6-week-old female C57BL/6 mice (weight: 18 g, n = 36) were provided by Hangzhou Medical College (China). These mice were firstly placed in a specific pathogen-free (SPF) environment for one-week adaptive feeding (23 °C ± 1 °C, Humidity: 50% ± 10%, a 12-h alternating light-dark cycle). Three mice were housed in each cage, with adequate food and clean water *ad libitum*. This animal study has been approved by the Committee of Zhejiang Baiyue Biotechnology Co., LTD. (ZJBYLA-IACUC-20240325), and the implementation of the study strictly adhered to relevant ethical standards.

### 2.2 Animal modeling and grouping

The experimental animals were divided into six groups using the random number table method: the control (Con), the model (Mod), the Mod + low-dose KF (KF-L), the Mod + medium-dose KF (KF-M), the Mod + high-dose KF (KF-H), and the Mod + mesalazine (mesa) groups (n = 6 mice/group). To induce chronic colitis (Wang et al., 2021), mice were treated with 2% dextran sulfate sodium (DSS) (0216011010, MP Biomedicals, United States) over multiple repeated cycles. In each cycle, mice were given 2% DSS in drinking water for 7 days, followed by sterile water for 14 days. The entire experiment lasted from day 1 to day 49 (Figure 1A). The mice in the control group received normal drinking water throughout the experiment. For the mice in the KF treatment groups, oral gavage of KF (HY-14590, MCE, United States) was initiated on the first day of DSS treatment, with daily doses of 25 mg/kg (low), 50 mg/kg (medium), or 100 mg/kg (high) (Qu et al., 2021). The mice in the positive control group received 200 mg/kg of mesalazine (HY-15027, MCE, United States) intragastrically every day (Figure 1A). During the treatment period, the body weight was recorded every 3 days. After treatment, the mice were dissected under anesthesia with pentobarbital sodium (STY667, Zzsiji, China) to collect colon tissues. The tissues were measured for length, and preserved for further testing. All mice were then euthanized by cervical dislocation under anesthesia.

**FIGURE 1:**
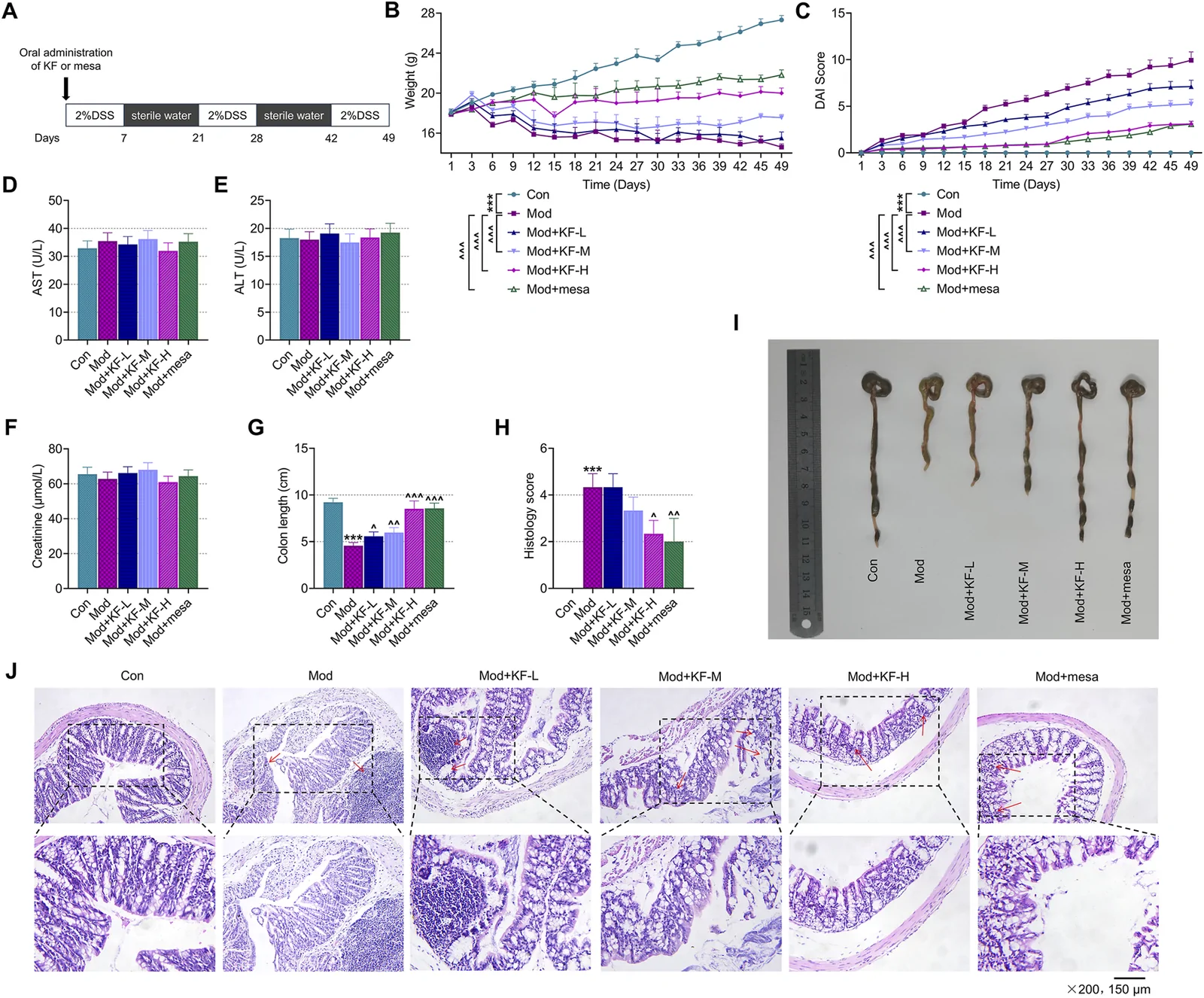
Kaempferol (KF) improved the pathological changes of mice with chronic colitis. **(A)** Detailed process and dosage regimen of DSS, KF and mesalazine (mesa). **(B)** The weight changes of mice with chronic colitis within 49 days (each group: n = 6). **(C)** Disease activity index was evaluated as the combined score of weight loss, fecal consistency and fecal blood (scored as 0–12). **(D–F)** Serum liver and kidney function tests (each group: n = 6). **(G,I)** Measurement of colon length in each group of mice (each group: n = 6). **(H,J)** Pathological changes in colon tissues were detected by Hematoxylin and Eosin (HE) staining (the red arrow indicates the site of inflammation, magnification: 200×, scale bars: 150 µm) (each group: n = 6). ^***^ *P* < 0.001 vs. Control (Con); ^^^ *P* < 0.05, ^^^^ *P* < 0.01, ^^^^^ *P* < 0.001 vs. Model (Mod).

Daily assessments of body weight, stool characteristics and bleeding status were performed during the experiment to evaluate the disease activity index (DAI). Briefly, DAI scores were defined as follows, for weight loss, 0: no loss; 1: 1%–5% loss; 2: 5%–10% loss; 3: 10%–20% loss and 4: >20% weight loss; for stool consistency, 0: normal; 2: loose stool; 4: diarrhea; and for stool bleeding, 0: no blood; 2: presence and 4: gross blood (Zhang et al., 2022).

### 2.3 Cell culture and grouping

Human colon cancer cells HT29 were obtained from Shanghai Anwei Biotechnology Co., Ltd. (AW- CELLS-H0161, China). Human colon tissue cells CCD-18Co (CL-0591) were purchased from Procell Life Science & Technology Co., Ltd. Cells were identified using short tandem repeat analysis. HT29 cells were cultured with McCOY’s5A (PM150710, Procell, China) + 10% fetal bovine serum (FBS, 10100139, ThermoFisher, United States) + 1% penicillin/streptomycin (P/S, C0222, Beyotime, China). CCD-18Co cells were incubated with Minimum Essential Medium (MEM, PM150410, Procell, China) + 10% FBS + 1% P/S. Cell incubation was performed at 37 °C with 5% CO_2_ (Forma Steri-Cult, ThermoFisher, United States).

In the cell experiments, the toxicity of different doses of KF (5, 10, 20 μM) on HT29 cells was detected (Tie et al., 2021), and ultimately 20 μM was selected for subsequent experimental research. First, the cells were divided into the control (normal culture), tumor necrosis factor α (TNF-α, HY-P70426G, MCE, United States) (culture in 100 ng/mL TNF-α (Drygiannakis et al., 2013)), KF (culture in 20 μM KF) and TNF-α +KF (culture in 100 ng/mL TNF-α and 20 μM KF) groups. Cells were collected after 48 h of culture. In the second part of the cell experiment, CCD-18Co cells were co-cultured with HT29 medium containing TNF-ɑ/KF/TNF-ɑ+KF for 48 h to detect cell viability and the levels of related proteins.

Next, the HT29 cell line with HMOX1 overexpression/knockdown was established. The short hairpin RNA targeting HMOX1 (sh-HMOX1, pLV [shRNA]-EGFP/Puro-U6>hHMOX1 [shRNA#1]) and its negative control (short hairpin RNA negative control, sh-NC), as well as HMOX1 expression lentiviral vector (OE-HMOX1, pLV [Exp]-Puro-UBC > hHMOX1 [NM_002133.3](ns):P2A:Luc2) and its negative control (overexpression negative control, OE-NC) were procured from Yunzhou Biosciences (Guangzhou) Co., Ltd. After 48 h of transfection with these plasmids, the transfection efficiency was evaluated using quantitative real-time polymerase chain reaction (qRT-PCR)/western blot. CCD-18Co cells were assigned into six groups, namely, TNF-α, TNF-α+KF, TNF-α+KF + OE-NC + shNC, TNF-α+KF + sh-HMOX1, TNF-α+OE-HMOX1 and TNF-α+KF + OE-HMOX1. HT29 cells in the TNF-α and TNF-α+KF groups were intervened with TNF-α/TNF-α+KF. The cells in the TNF-α+KF + OE-HMOX1, TNF-α+KF + OE-NC + shNC and TNF-α+KF + sh-HMOX1 groups were transfected with HMOX1 overexpression plasmid/sh-HMOX1/negative control and then intervened with TNF-α+KF. The cells in the TNF-α+OE-HMOX1 group were treated with TNF-α after transfection with HMOX1 overexpression treatment. The duration of intervention with TNF-α/KF/TNF-α +KF in each group was 48 h. Then, the culture media of each group were collected and co-cultured with CCD-18Co cells for 48 h, followed by cell viability detection and protein determination.

In the third part of the cell experiment, CCD-18Co cells were treated with TNF-α/KF/TNF-ɑ+KF, following the same procedures as described in the previous cell experiments. Then the culture medium was collected and co-cultured with HT29 cells for 48 h. Finally, cell viability and protein levels were assessed.

### 2.4 Serum biochemical analyses

The ALT, AST, Creatinine levels were determined in the serum using the appropriate test kits (E-BC-K235-M, E-BC-K236-M, Elabscience, than, China; ZK-5450, ChemicalBook, China). In brief, the indicated concentration of mice serum was added into the enzyme-labelled plates and incubated at 37 °C for 120 min. Then, the supernatant in the plates was discarded, 100 μL biotinylated antibody was added, and the incubation was continued for 60 min. The plates were washed with the buffer solution thrice. Subsequently, 100 μL streptavidin-HRP working solution was added and incubated at 37 °C for 30 min. Following these, the TMB substrate were put into plates and incubated at 37 °C for 15 min in dark. Finally, 50 μL stop solution was added, and the OD values were measured by using a microplate reader (1,510, Thermo Fisher Scientific, MA, United States) at 450 nm.

### 2.5 Hematoxylin and eosin (HE) staining

Colon tissues were fixed in 4% paraformaldehyde (P0099, Beyotime, Shanghai, China), and then dehydrated successively with 70/80/95% ethanol and anhydrous ethanol (C06915101, Nanjing Reagent, China). After being transparentized with xylene (C04305302, Nanjing Reagent, China), the tissues were embedded in paraffin, sliced, dewaxed with xylene, and hydrated with ethanol of different concentrations. Following staining with hematoxylin (C0107, Beyotime, China) and eosin (C0109, Beyotime, China), the sections were again dehydrated in ethanol, permeabilized with xylene, and finally mounted with neutral gum (C0173, Beyotime, China). Colonic tissue lesions were detected under a microscope (200×, Ts2R-FL, Nikon, Japan), and scored for severity (Wu et al., 2026).

### 2.6 Masson staining

Colon tissues were fixed with 4% paraformaldehyde, dehydrated with gradient ethanol, transparentized with xylene, then embedded in paraffin and sectioned. After the sections were dewaxed with xylene and hydrated with ethanol, they were stained with Masson’s three-color staining solution (C0189S, Beyotime, China), including hematoxylin, Lichun Red acidic fuchsin solution, etc. Following a series of ethanol dehydration, xylene transparency, and neutral gum sealing, the colonic fibrosis and collagen deposition were observed under a microscope (200×) and quantified analyzed was measured with ImageJ software (National Institute of Health, Bethesda, Md.).

### 2.7 Western blot

Proteins were extracted from tissues or cells using radioimmunoprecipitation assay buffer (RI-PA) lysate (R0010, Solarbio, China). The bicinchoninic acid assay (BCA, BCA1, Sigma-Aldrich, United States) was used to measure the protein concentration. Proteins were separated by sodium dodecyl sulfate-polyacrylamide gel electrophoresis (SDS-PAGE, P0012A, Beyotime, China), and wetly transferred to polyvinylidene fluoride (PVDF) membrane (YA1700, Solarbio, China). The 5% skim milk was allowed to equilibrate at room temperature for 1.5 h for sealing the proteins. before the monoclonal dilution was added. Next, the membrane was cultivated with primary antibody at 4 °C overnight. The next day, the membrane was incubated with the corresponding secondary antibody for 2 h. Protein bands were visualized using the gel imaging device (ChampGel®7800, SINSAGE, China). The intensity of the protein bands was quantified using ImageJ software (1.54p, National Institutes of Health, United States). Original blot images were converted to 8-bit grayscale format. The integrated optical density (IOD) of each band was measured after subtracting the local background intensity. To normalize for protein loading variations, the IOD of the target protein was divided by the IOD of the corresponding loading control (GAPDH). The relative expression levels represent the mean ± SD from at least three independent experiments. The antibody information was listed in Table 1.

**TABLE 1:** Antibodies used in this study.

Name	Catalog	Molecular weight	Dilution	Manufacturer
TNF-α	ab6671	30 kDa	1/1,000	Abcam, United Kingdom
ZO-1	ab276131	195 kDa	1/1,000	Abcam, United Kingdom
α-SMA	ab7817	40 kDa	1 μg/mL	Abcam, United Kingdom
Col1a1	ab6308	139 kDa	1/7500	Abcam, United Kingdom
HMOX1	ab189491	33 kDa	1/2000	Abcam, United Kingdom
MMP2	ab92536	74 kDa	1/1,000	Abcam, United Kingdom
MMP9	30592-1-AP	105 kDa	1/2000	Proteintech, China
NRF2	ab325240	110 kDa	1/1,000	Abcam, United Kingdom
LaminB1	ab16048	66 kDa	1/1,000	Abcam, United Kingdom
GAPDH	ab8245	36 kDa	1/10,000	Abcam, United Kingdom
Goat anti rabbit	ab205718	—	1/2,000	Abcam, United Kingdom
Goat anti mouse	ab205719	—	1/2,000	Abcam, United Kingdom

### 2.8 Transepithelial electrical resistance (TER) of colonic epithelial

Colonic epithelial permeability was investigated using an Ussing chamber (Physiologic Instrument, United States). The distal colon was longitudinally divided and tissue was pinned on a 0.3 cm^2^ disk. The disk was inserted into the Ussing chamber, which contained oxygenated Krebs solution at 37 °C. TER was measured when a current of 3 µA was passed. TEER was calculated according to Ohm’s law (Srinivasan et al., 2015).

### 2.9 QRT-PCR

Trizol reagent (R0016, Beyotime, Shanghai, China) was used to isolate total RNAs from tissues or cells. Then, cDNA was synthesized using the BeyoRT™ II cDNA First Strand Synthesis Kit (D7168S, Beyotime, Shanghai, China). QRT-PCR was performed employing the BeyoFast™ Probe qPCR Mix (D7271, Beyotime, Shanghai, China) and a PCR instrument (CFX96, Bio-rad, United States). The results were normalized to glyceraldehyde -3-phosphate dehydrogenase (GAPDH), and the 2^−ΔΔCt^ method was used to determine the mRNA expression levels. The primer sequences (Table 2) were produced by Tsingke (Beijing, China).

**TABLE 2:** Gene sequence primers.

Name	Forward primer (5′-3′)	Reverse primer (5′-3′)
HMOX1	CCT​CAC​AGA​TGG​CGT​CAC​TT	TGG​GGG​CCA​GTA​TTG​CAT​TT
COL1A1	CCT​GTC​TGC​TTC​CTG​TAA​ACT	TCC​AGG​AGC​ACC​AAC​ATT​AC
COL3A1	TTC​TCG​CTC​TGC​TTC​ATC​CC	TCC​GCA​TAG​GAC​TGA​CCA​AG
FN1	CTG​AGA​CCA​TCA​CCA​TTA​G	GGG​CTC​GCT​CTT​CTG​ATT​ATT
GAPDH	GAC​AAG​ATG​GTG​AAG​GTC​GGT	CTT​TGG​CAT​CGT​GGA​AGG​GCT​C

### 2.10 T-AOC, GSH-Px, SOD and MDA assessment

The levels of T-AOC (Beyotime, Beijing, S0119), GSH-Px (Beyotime, Beijing, S0057S), SOD (Beyotime, Beijing, S0109) and MDA (Beyotime, Beijing, S0131S) in lysed colon samples were measured using corresponding commercial kits according to the manufacturers’ instruction. The bicinchoninic acid assay (BCA, BCA1, Sigma-Aldrich, United States) was used to measure the protein concentration.

### 2.11 Intracellular reactive oxygen species (ROS) generation

HT29 cells were seeded in 24-well plates and incubated overnight. Subsequently, fluorescent probes (DCFH-DA, DHE, and DAF-FM DA) were added into each well. After staining for 30 min, cells were washed with PBS, and their nuclei were stained by DAPI for 20 min. Finally, the fluorescence intensities of cells were analyzed by FCM (ACEA Novocyte™) based on 10,000 gated cell events.

### 2.12 Methylthiazolyldiphenyl-tetrazolium bromide (MTT) experiment

Cells were inoculated in 96-well plates (167008, Thermo Fisher, United States) (5,000 cells/well in 100 µL). After 24 h of culture, cells were treated with KF (5, 10, 20 μM) or left untreated (control) and cultured for 48 h. Then 10 µL of MTT solution (C0009S, Beyotime, China) was added per well for 4 h, followed by 100 µL of formazan dissolution solution for 3–4 h. The absorbance values of each well were measured at a wavelength of 570 nm using an enzyme reader (Varioskan LUX, Thermo Fisher, United States) to evaluate the cytotoxicity of KF.

### 2.13 Enzyme-linked immunosorbent assay (ELISA)

Cell culture medium samples were collected. According to the instructions of the ELISA kit (E-EL-M0037, E-EL-M0044, E-EL-M3063, E-EL-H0149, E-EL-H6156, E-EL-H0109, E-EL-0162, E-EL-H2172, E-EL-H0084, Elabscience, China), samples were added sequentially, followed by the incubation with biotinylated antibody working solution and plate washing. Then, the samples were cultivated with enzyme binding working solution, washed, and colored by substrate solution. The reaction was terminated with the stop solution. The absorbance was measured at 450 nm on the microplate reader, and the level of IL-1β, IL-6, TNF-α, TGF-β1, HO-1 and hepatocyte growth factor (HGF) in the culture medium was calculated through the standard curve.

### 2.14 Cell counting kit-8 (CCK-8)

Cells in the logarithmic growth phase were seeded into 96-well plates at a density of 5,000 cells/well and cultured overnight in a CO_2_ incubator. After cell adhesion, the experimental groups were treated with the designated interventions, while the control group received double-distilled water. Each group had six replicate wells. Following 24-h incubation, 10 µL of CCK-8 reagent (C0038, Beyotime, China) was added to each well for 2-h culture. Absorbance at 450 nm was measured using a microplate reader.

### 2.15 Statistical analysis

Statistical analyses were performed using GraphPad Prism 8.0. The Shapiro-Wilk test was used to check for normality. Two-factor analysis of variance was employed for comparisons across several time periods (Figure 1B). One-way analysis of variance (ANOVA) was used for comparison among several groups, followed by Tukey’s *post hoc* test. The primary endpoints of this study were predefined as the disease activity index (DAI) score and histological damage score, which were assessed in a blinded manner throughout the experimental period. Secondary endpoints included fibrosis, pro-inflammatory cytokine levels, and expression of HMOX1 and related signaling molecules. *In vivo* experiments, a minimum of six animals per group was calculated based on a large effect size and statistical power (Power = 0.8, α = 0.05, Cohen’s d ≈ 2.1) observed in our preliminary pilot study. For *in vitro* experiments, data are presented as the mean ± SD of at least three independent biological replicates (n = 3), which is standard practice to ensure reproducibility. These sample sizes were deemed adequate to detect statistically significant differences. A *P* value < 0.05 was considered statistically significant.

## 3 Results

### 3.1 KF treatment alleviated chronic colitis in mice

First of all, we observed the changes in the body weight and DAI score of mice. Compared with the control group, the body weight of the mice was decreased rapidly and an elevated DAI score (Figures 1B,C, *P* < 0.001). Treatment with mesalazine or KF-M/H (50/100 mg/kg) resulted in an apparent increased body weight and decreased DAI scores (Figures 1B,C, *P* < 0.001), but KF-L (25 mg/kg) had no significant effect on the body weight or DAI scores of model mice (Figures 1B,C). After the experiment was completed, the changes in AST, ALT and creatinine levels in mice across different groups demonstrated that KF exhibited no hepatotoxic or nephrotoxic effects (Figures 1D–F). After modeling, the colon length was significantly shortened (Figures 1G,I, *P* < 0.001). In all treatment groups, the colonic length demonstrated varying degrees of improvement (Figures 1G,I, Mod vs. Mod + KF-L *P* = 0.0350). The results of colonic tissue pathological staining showed normal structure of colonic tissue and orderly cell arrangement in the control group (Figures 1H,J). In contrast, the modeling led to disrupted tissue structure, abnormal cell morphology, and pronounced inflammatory lesions, with a tissue score significantly higher than that of the control group (Figures 1H,J, *P* < 0.001). Compared with the model group, each treatment group showed variable improvement in pathological conditions, with tissue structure approaching normal and cell arrangement becoming relatively regular (Figures 1H,J). However, only KF-H and mesalazine treatments significantly reduced the tissue score (Figures 1H,J, Mod vs. Mod + KF-H *P* = 0.0191, Mod vs. Mod + mesa *P* = 0.0064).

### 3.2 KF exerts a protective effect against histopathological injury and oxidative stress in mouse colon tissue

According to Masson staining data, the colon tissue structure of the control group was normal and the collagen fibers were evenly distributed (Figure 2A). Obvious collagen deposition were observed in the model group, with disordered tissue structure and a significantly elevated CVF (Figures 2A,B *P* < 0.001). Compared with the model group, treatment groups presented reduced area of collagen deposition, and apparently restored tissue structure (Figures 2A,B, *P* < 0.001). Furthermore, The levels of inflammatory cytokines IL-1β, IL-6 and TNF-α in mouse blood were significantly upregulated due to modeling (Figures 2C–E, *P* < 0.01). In contrast, the KF-L/H/M group and the mesalazine-treated group exhibited significantly reduced levels of these inflammatory cytokines (Figures 2C–E, *P* < 0.01). In the model group, the expressions of TNF-α and alpha smooth muscle actin (α-SMA) in the colon tissue were significantly upregulated, and ZO-1 was downregulated (Figures 2F–I,P < 0.001). After treatment, TNF-α and α-SMA level in KF-H/M and mesalazine treatment groups were both lower than those in the model group (Figures 2F–I,P < 0.05). Except that KF-L barely affected ZO-1 expression, the expression was upregulated in other treatment groups (Figures 2F,H, *P* < 0.01). We also measured intestinal cell TER. In the model group, TER was significantly reduced (Figure 2J, *P* < 0.001), whereas in all treatment groups, TER was significantly increased (Figure 2J, *P* < 0.001). Meanwhile, both the KF-H group and mesalazine treatment reduced the elevated expression of collagen type I alpha 1 chain (Col1a1) induced by the modeling procedure (Figure 3A, Mod vs. Mod + KF-H *P* =0.0073).

**FIGURE 2:**
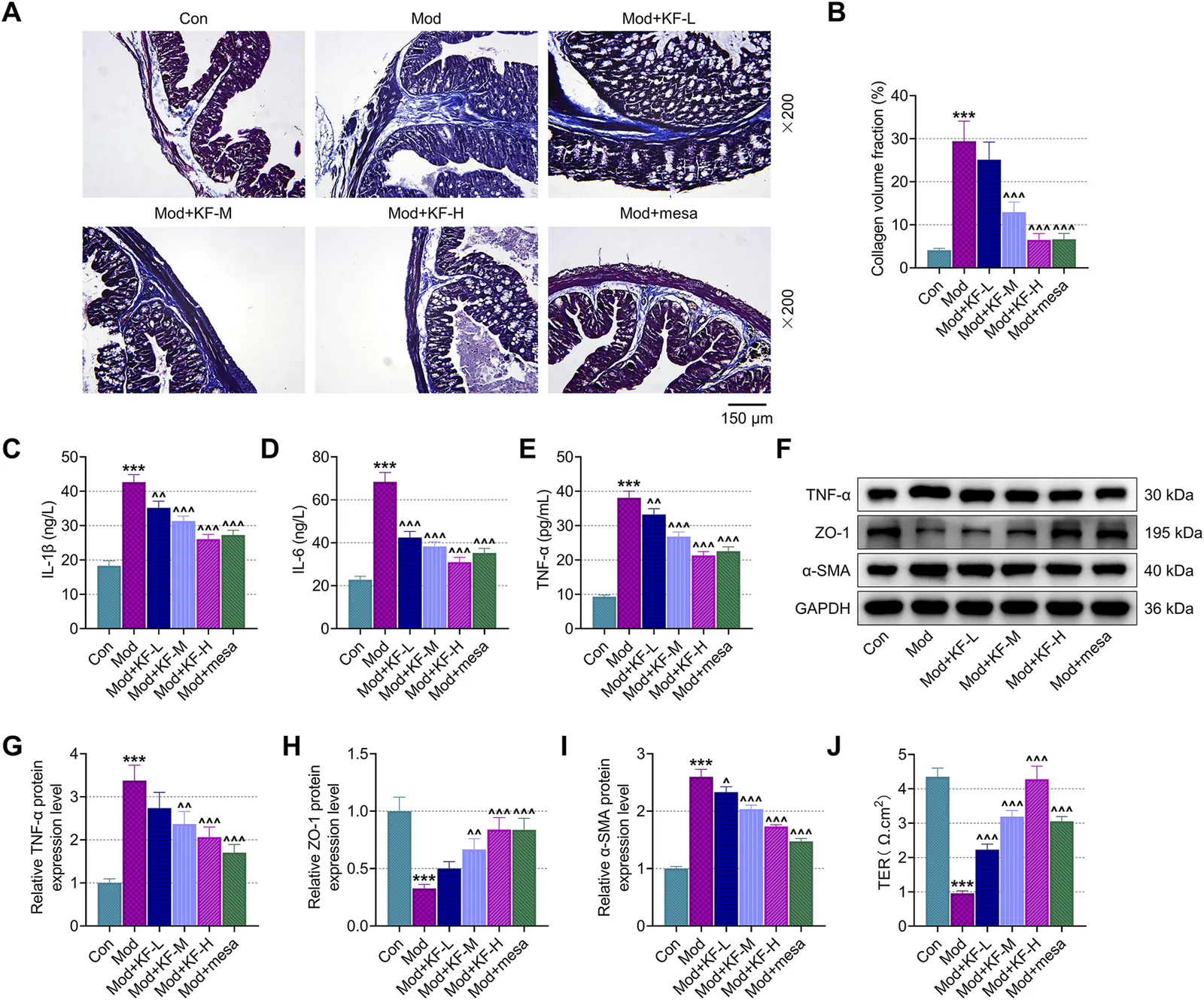
KF alleviated fibrosis and inflammatory cytokines in mice. **(A,B)** Masson staining was used to detect colonic fibrosis and collagen deposition (magnification: 200×, scale bars: 150 µm) (each group: n = 6). **(C–E)** ELISA assay for determining the levels of inflammatory cytokines (each group: n = 6). **(F–I)** The expression levels of tumor necrosis factor α (TNF-α), zonula occludens-1 (ZO-1) and alpha smooth muscle actin (α-SMA) in colon tissues were measured by western blot. Glyceraldehyde -3-phosphate dehydrogenase (GAPDH) was the internal parameter (each group: n = 6). **(J)** Transepithelial electrical resistance the Ussing chamber experiment (each group: n = 6). ^***^ *P* < 0.001 vs. Con; ^^^ *P* < 0.05, ^^^^ *P* < 0.01, ^^^^^ *P* < 0.001 vs. Mod.

**FIGURE 3:**
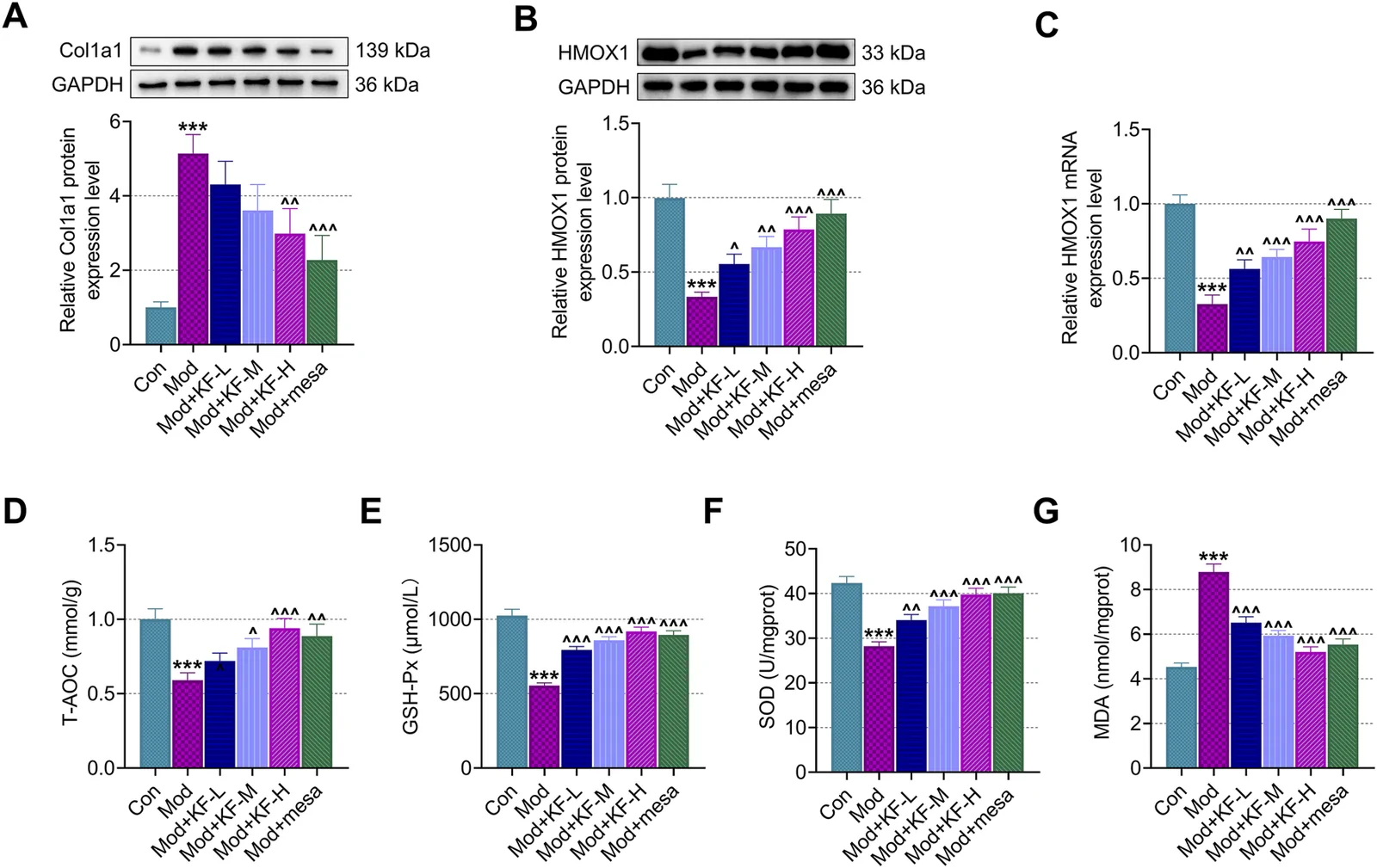
KF reduces levels of Col1a1 protein and oxidative stress. **(A)** The expression levels of collagen type I alpha 1 chain (Col1a1) in colon tissues were measured by western blot. GAPDH was the internal parameter (each group: n = 6). **(B)** The level of heme oxygenase 1 (HMOX1) in colon tissue was detected by western blot. GAPDH was the internal parameter (each group: n = 6). **(C)** The level of heme oxygenase 1 (HMOX1) in colon tissue was detected by quantitative real-time polymerase chain reaction (qRT-PCR). GAPDH was the internal parameter (each group: n = 6). **(D–G)** The level of total antioxidant capacity (T-AOC), glutathione peroxidase (GSH-Px), superoxide dismutase (SOD) and malondialdehyde (MDA) in colon tissue was detected by commercially available kits (each group: n = 6). ^***^ *P* < 0.001 vs. Con; ^^^ *P* < 0.05, ^^^^ *P* < 0.01, ^^^^^ *P* < 0.001 vs. Mod.

HMOX1 level in the colon tissue of mice was significantly downregulated after modeling, which was reversed by different doses of KF or mesalazine treatment (Figures 3B,C, *P* < 0.05). We also measured the levels of oxidative stress in colonic tissue. KF or mesalazine increased T-AOC (Mod vs. Mod + KF-H *P* = 0.0003), GSH-Px, and SOD (Mod vs. Mod + KF-L *P* = 0.0019), which were reduced by DSS (Figures 3D–F, *P* < 0.05), and decreased MDA, which was elevated by DSS (Figure 3G, *P* < 0.001). These findings demonstrate that KF suppresses intestinal inflammatory responses and oxidative stress, inhibits the fibrotic process in the mouse intestine, and promotes the repair of the intestinal epithelial barrier.

### 3.3 KF treatment alleviated colonic epithelial cell inflammation caused by pro-inflammatory cytokine intervention

In this part of the experiment, we first detected the toxicity of different concentrations of KF on HT29 cells and found that 5, 10, and 20 μM had no significant toxicity (Figure 4A). Subsequently, HT29 cells were intervened with TNF-α and/or KF (20 μM). We found that the protein levels of ZO-1 and HMOX1 in the cells were evidently diminished after TNF-α intervention, which was counteracted by KF treatment (Figures 4B–D, *P* < 0.05). Besides, TNF-α treatment elevated the level of TGF-β1 and HO-1, but subsequent treatment of KF reduced its level (Figures 4E,F, HO-1: TNF-α vs. TNF-α+KF *P* = 0.0009). The HMOX1-mediated antioxidant effect is closely linked to the activation of the NRF2 pathway. KF treatment does not enhance NRF2 nuclear translocation (Figures 4G,H); however, following TNF-α treatment, KF therapy significantly enhances NRF2 nuclear translocation (Figures 4G,H, *P* < 0.01). Subsequently, we measured the levels of ROS and SOD activity in HT29 cells. TNF-α treatment increased ROS levels and decreased SOD activity (Figures 4I,J, *P* < 0.001). However, KF was able to mitigate the oxidative cellular damage induced by TNF-α (Figures 4I,J, *P* < 0.01). In contrast, KF treatment alone had no significant effect on the levels of ROS and SOD activity (Figures 4I,J). These findings suggest that the antioxidant effect of KF is mediated indirectly through the Nrf2/HMOX1 axis.

**FIGURE 4:**
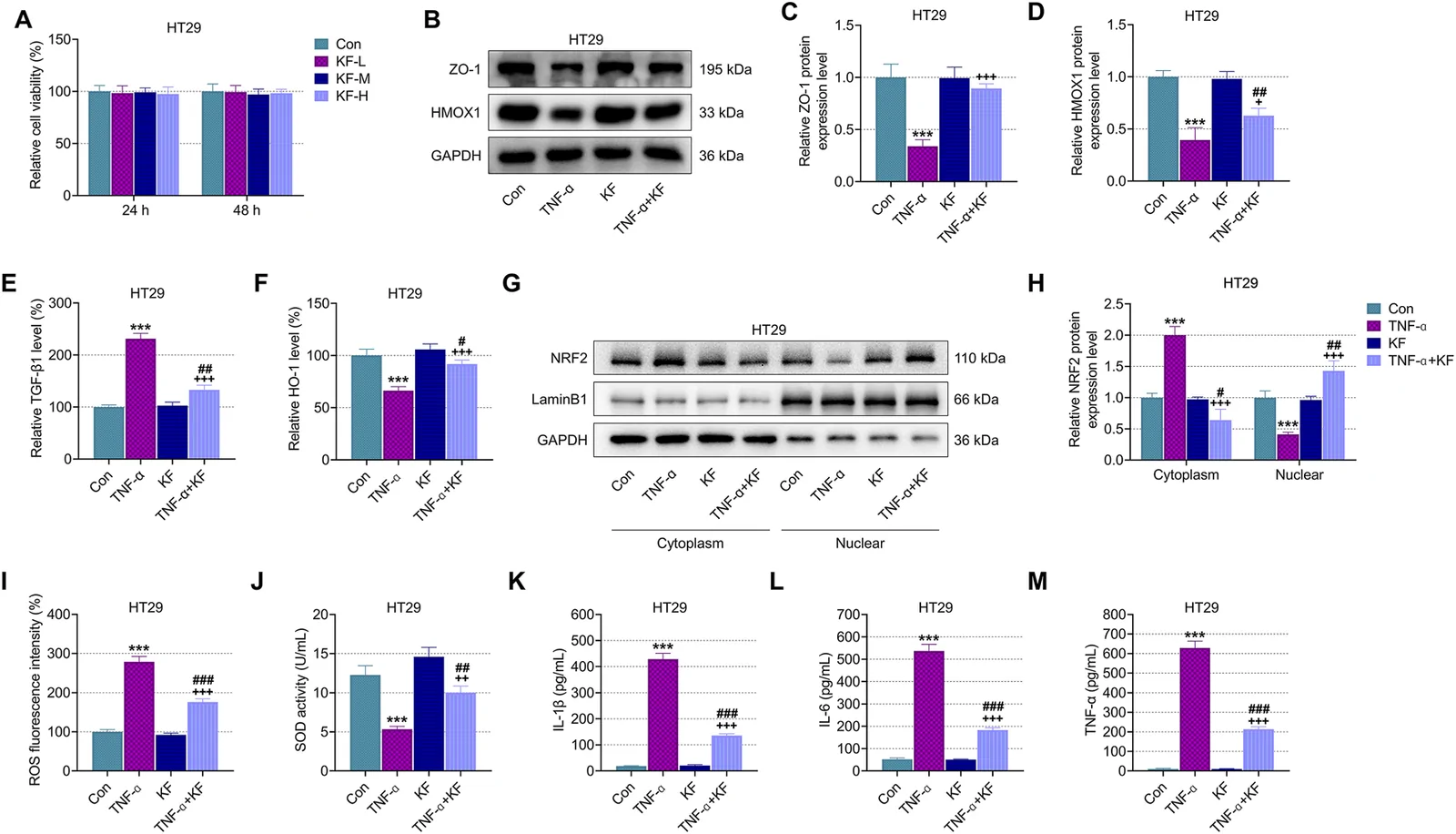
KF regulated HT29 cell inflammation and oxidative stress induced by TNF-α intervention. **(A)** Methylthiazolyldiphenyl-tetrazolium bromide (MTT) was employed to determine the cytotoxicity of KF against HT29 cells. **(B–D)** The protein expression levels of ZO-1 and HMOX1 in each group of cells were assessed by western blot. GAPDH was the internal parameter. **(E,F)** The level of transforming growth factor-β1 (TGF-β1) and HO-1 in the cell culture medium was detected by enzyme-linked immunosorbent assay (ELISA). **(G,H)** Western blot was used to examine the NRF2 nuclear translocation. GAPDH, LaminB1 was the internal parameter. **(I)** Flow cytometry-based detection of intracellular reactive oxygen species (ROS) generation. **(J)** The level of SOD was detected by commercially available kits. **(K–M)** ELISA assay for determining the levels of inflammatory cytokine (IL-1β, IL-6 and TNF-α). Each experiment was repeated 3 times. ^**^ *P* < 0.01, ^***^ *P* < 0.001 vs. Con; ^+^ *P* < 0.05, ^+++^ *P* < 0.001 vs. TNF-α; ^#^ *P* < 0.05, ^##^ *P* < 0.01, ^###^ *P* < 0.001 vs. KF.

### 3.4 KF suppresses fibroblast activation and the expression of fibrosis-related genes through a co-culture system

Then we co-cultured the HT29 medium containing TNF-α/TNF-α+KF with CCD-18Co cells. TNF-α treatment significantly induced elevated levels of IL-1β, IL-6 and TNF-α in the HT29 cell supernatant (Figures 4K–M, *P* < 0.001). KF significantly reduced these inflammatory cytokine levels (Figures 4K–M, *P* < 0.001). The cell viability detection experiment revealed that CCD-18Co cell viability was enhanced by TNF-α, but later inhibited by KF intervention (Figure 5A, TNF-α vs. TNF-α+KF *P* = 0.0136). Next, we quantified the activation markers of CCD-18Co cells, and confirmed that TNF-α augmented MMP2/9 and α-SMA protein levels in CCD-18Co cells, which was offset by KF treatment (Figures 5B–E, *P* < 0.05). qRT-PCR analysis revealed that TNF-α also upregulated the expression of *Col1a1, Col3a1* and *FN1* in CCD-18Co cells, whereas KF treatment abolished this effect (Figures 5F–H, *P* < 0.001). These results demonstrate that KF inhibits fibroblast activation.

**FIGURE 5:**
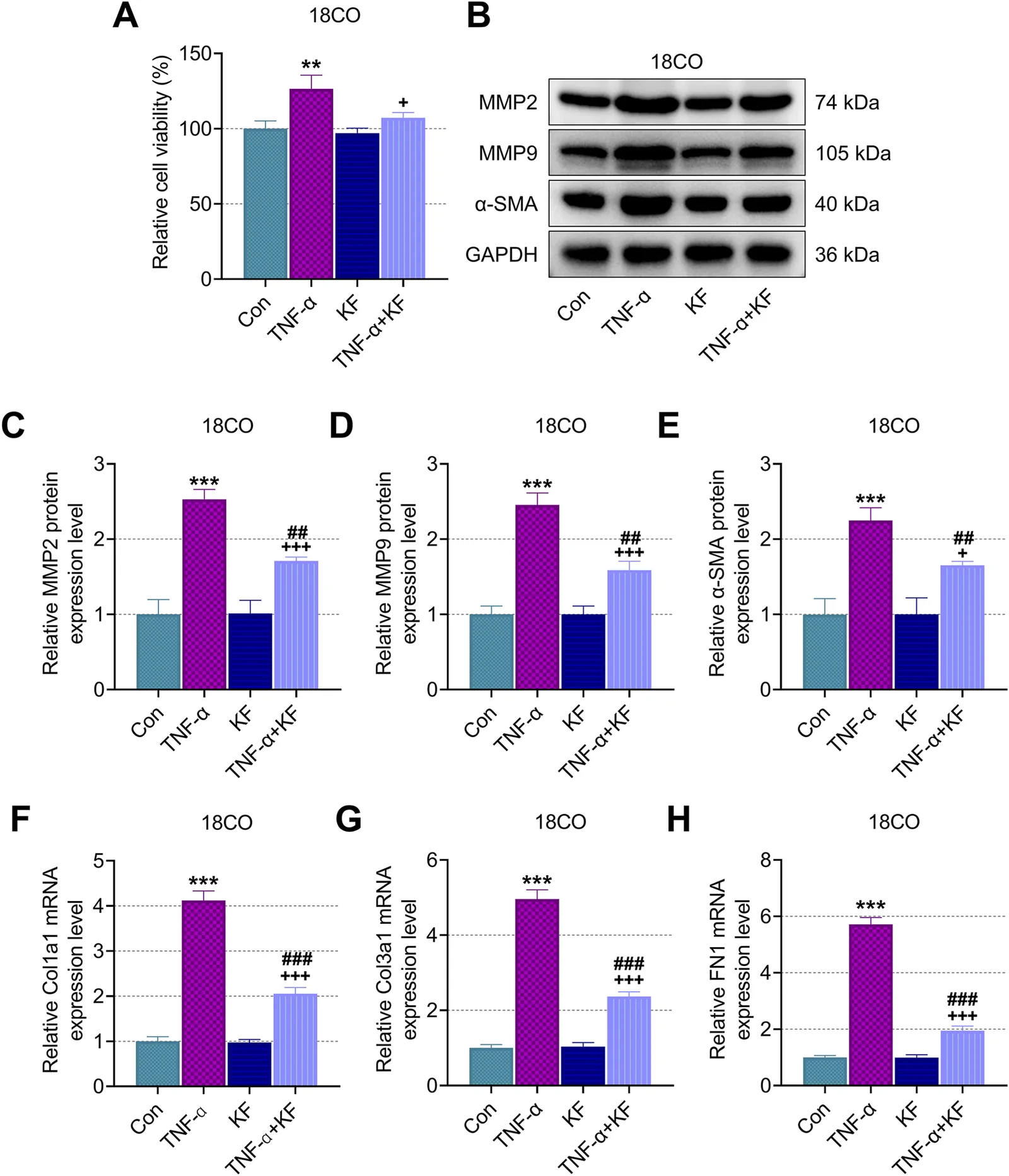
KF regulation of TNF-α-induced CCD-18Co cell activation. **(A)** The viability of CCD-18Co cells was tested by cell counting kit-8 (CCK-8). **(B–E)** Western blot was used to examine the levels of activation marker proteins in CCD-18Co cells, including matrix metallopeptidase (MMP) 2/9 and α-SMA. GAPDH was the internal parameter. **(F–H)** The level of FN1, Col1a1 and Col3a1 was detected by qRT-PCR. Each experiment was repeated 3 times. ^**^ *P* < 0.01, ^***^ *P* < 0.001 vs. Con; ^+^ *P* < 0.05, ^+++^ *P* < 0.001 vs. TNF-α; ^##^ *P* < 0.01, ^###^ *P* < 0.001 vs. KF.

### 3.5 Regulation of HMOX1 reversed the inhibitory effect of KF on fibroblast activation

Then we successfully established HT29 cells with HMOX1 silencing/overexpression (Figures 6A–C, *P* < 0.001). After transfection with sh-HMOX1/HMOX1 overexpression plasmid, HT-29 cells were exposed to TNF-α/TNF-α+KF, and then the culture medium was collected for co-culture with CCD-18Co cells. The results indicated that compared with that in the TNF-α group, the cell viability in the TNF-α+KF group was decreased significantly, but HMOX1 knockdown reversed the inhibiting role of KF in cell viability (Figure 6D, *P* < 0.05). We also proved that relative to those in the TNF-α group, the activation marker proteins MMP2/9 and α-SMA in the TNF-α +KF group were notably downregulated, which was reversed by HMOX1 knockdown (Figures 6E–H, *P* < 0.05). Moreover, KF combined with HMOX1 overexpression exerted more obvious effects on suppressing these proteins (Figures 6E–H, *P* < 0.05). The NF-α + KF group also exhibited downregulation of *Col1a1, Col3a1* and *FN1* mRNA expression (Figures 6I–K, *P* < 0.001). Knockdown of HMOX1 reversed this effect, whereas HMOX1 overexpression promoted it (Figures 6I–K, *P* < 0.05).

**FIGURE 6:**
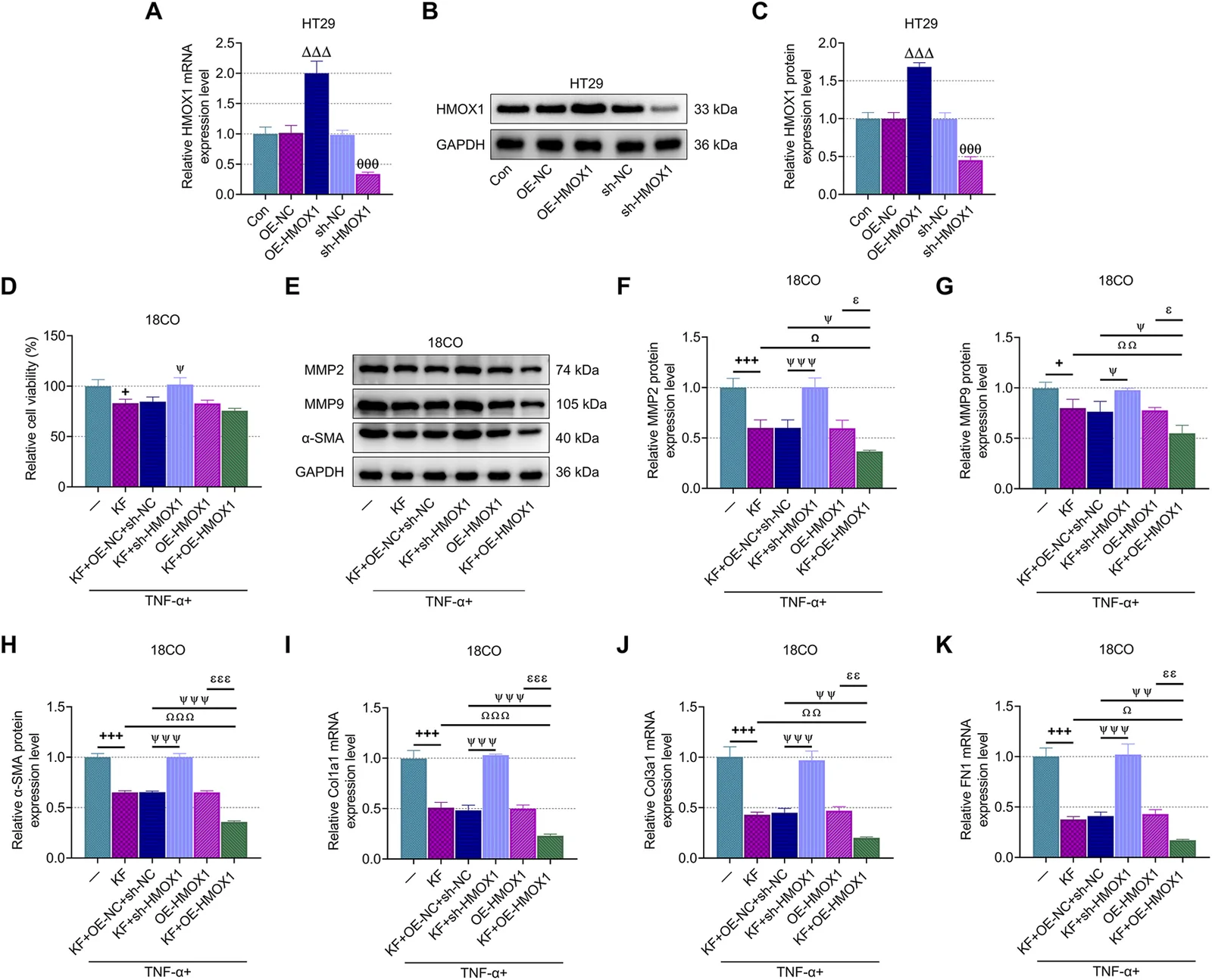
HMOX1 knockdown reversed the inhibiting effect of KF on TNF-α-induced activation of CCD-18Co cells. **(A–C)** The transfection efficiency of sh-HMOX1/HMOX1 overexpression plasmids was detected by qRT-PCR/western blot. GAPDH was the internal parameter. **(D)** The viability of CCD-18Co cells was examined by CCK-8. **(E–H)** Western blot was used to quantify the levels of activation marker proteins in CCD-18Co cells, including MMP2/9 and α-SMA. GAPDH was the internal parameter. **(I–K)** The level of FN1, Col1a1 and Col3a1 was detected by qRT-PCR. Each experiment was repeated 3 times. ^ΔΔΔ^ *P* < 0.001 vs. overexpression negative control (OE-NC); ^θθθ^ *P* < 0.001 vs. short hairpin RNA negative control (sh-NC); ^+^ *P* < 0.05, ^+++^ *P* < 0.001 vs. TNF-α; ^ψ^ *P* < 0.05, ^ψψ^ *P* < 0.01, ^ψψψ^ *P* < 0.001 vs. TNF-ɑ+KF + OE-NC + sh-NC; ^Ω^ *P* < 0.05, ^ΩΩ^ *P* < 0.01, ^ΩΩΩ^ *P* < 0.001 vs.TNF-ɑ+KF; ^ε^ *P* < 0.05, ^εε^ *P* < 0.01, ^εεε^ *P* < 0.001 vs. TNF-ɑ+overexpression (OE)-HMOX1.

We treated HT29 cells with the HO-1 inhibitor HO-1-IN-1 (10 µM), followed by collection of the culture supernatant for co-culture with CCD-18Co cells. Similarly, HO-1-IN-1 reversed the inhibitory effect of KF on cell viability (Figure 7A, KF vs. KF + HO-1-IN-1 *P* = 0.0238). Furthermore, HO-1-IN-1 significantly upregulated the expression of MMP2/9 (MMP2: KF vs. KF + HO-1-IN-1 *P* = 0.0092, MMP9: KF vs. KF + HO-1-IN-1 *P* = 0.0252) and α-SMA (KF vs. KF + HO-1-IN-1 *P* = 0.0130), which were downregulated by KF (Figures 7B–E).

**FIGURE 7:**
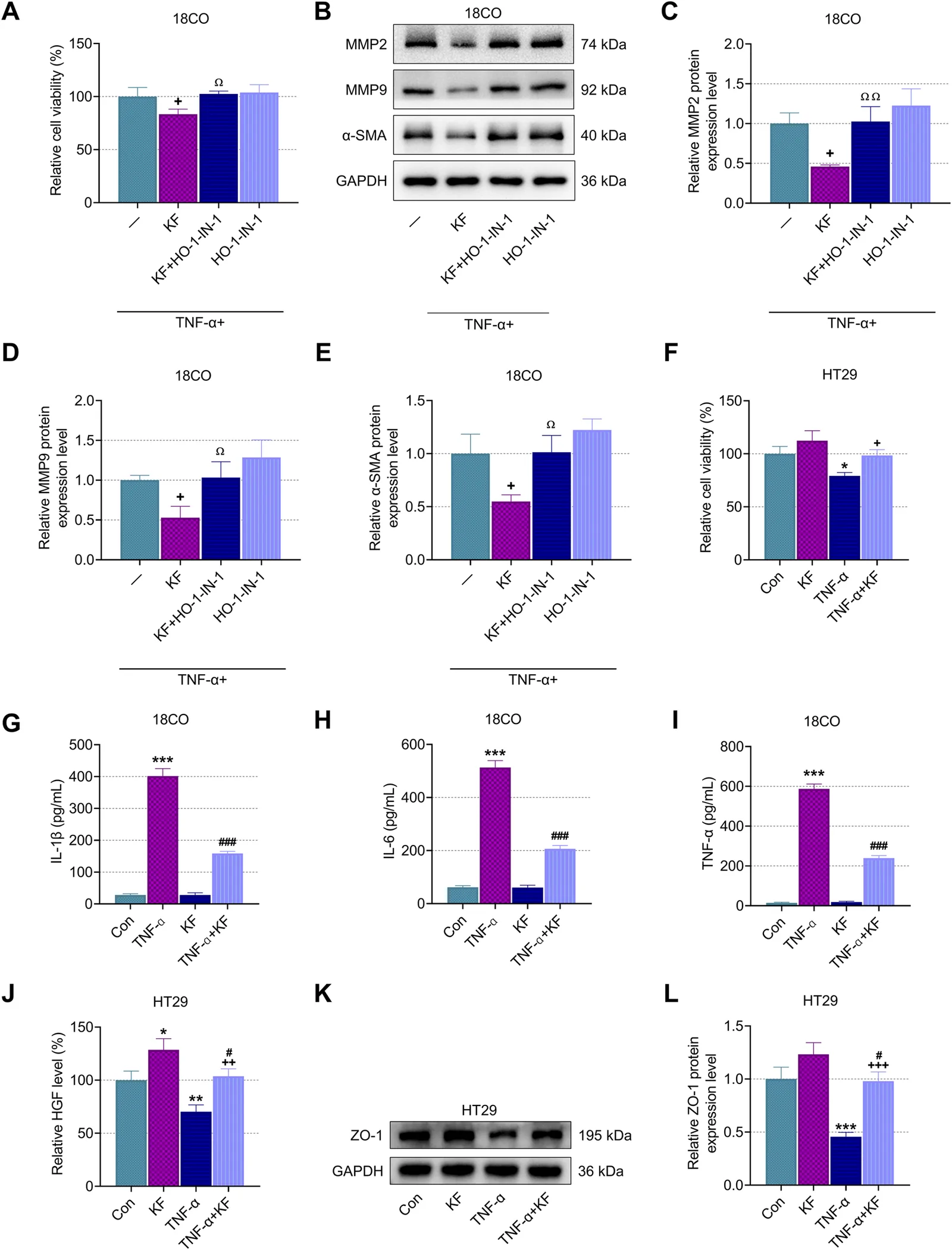
HO-1 inhibitors reverse the effects of KF, and KF can also influence HT29 cells in a co-culture system. **(A)** The viability of CCD-18Co cells was examined by CCK-8. **(B–E)** Western blot was used to quantify the levels of activation marker proteins in CCD-18Co cells, including MMP2/9 and α-SMA. GAPDH was the internal parameter. **(F)** The viability of HT-29 cells was detected by CCK-8. **(G–I)** ELISA assay for determining the levels of inflammatory cytokine (IL-1β, IL-6 and TNF-α). **(J)** ELISA was used to measure the level of hepatocyte growth factor (HGF) in the culture medium. **(K,L)** The protein level of ZO-1 in HT29 was tested by western blot. GAPDH was the internal parameter. Each experiment was repeated 3 times. ^+^ *P* < 0.05, ^++^ *P* < 0.01, ^+++^ *P* < 0.001 vs. TNF-α; ^Ω^ *P* <0.05 vs. TNF-α+KF; ^*^ *P* < 0.05, ^**^ *P* < 0.01, ^***^ *P* < 0.001 vs. Con; ^#^ *P* < 0.05, ^###^ *P* < 0.001 vs. KF.

### 3.6 KF protects HT29 cell viability and epithelial barrier function in a co-culture system

Next, we treated CCD-18Co cells with the same dose of TNF-α and/or KF, and then collected the culture medium to co-culture with HT-29 cells. We found that TNF-α treatment attenuated the promoting effect of fibroblasts on the activity of HT-29 cells, which was reversed by KF intervention (Figure 7F, TNF-α vs. TNF-α+KF *P* = 0.0305). Moreover, TNF-α treatment significantly increased the levels of IL-1β, IL-6 and TNF-α in the CCD-18Co cell supernatant, whereas KF treatment suppressed this inflammatory cytokines (Figures 7G–I,P < 0.001). Furthermore, TNF-α treatment dwindled HGF level, and KF treatment elevated and reversed the inhibition of TNF-α on HGF (Figure 7J, *P* < 0.05). Finally, we detected the protein level of ZO-1 in HT-29 cells and found that TNF-α treatment inhibited the protein level, which was counteracted by KF treatment (Figures 7K,L, *P* < 0.001).

## 4 Discussion

KF, as a flavonoid compound with multiple biological activities, plays an important role in various inflammatory diseases (Xiang et al., 2021; Wen et al., 2024). In atherosclerotic conditions, KF hinders disease progression by regulating macrophage function, and inhibiting lipid accumulation and inflammatory responses (Chu et al., 2024). The antioxidant and anti-inflammatory properties of KF are also critical in other inflammatory diseases. For instance, KF can protect mice with vascular injury from oxidative stress damage by regulating intracellular signaling pathways such as Nrf2/HMOX1, and reducing the production of inflammatory factors (Yao et al., 2020). In this study, the mechanism of KF on IBD further revealed its important position in inflammatory diseases.

In this study, KF treatment increased body weight and reduced colonic tissue damage in mice, indicating that KF can alleviate IBD by improving intestinal absorption, reducing inflammation, inhibiting inflammation and promoting tissue repair. Factors such as TNF-α and α-SMA play a key role in the occurrence and development of IBD. TNF-α serves as a main inflammatory mediator in the intestinal inflammatory process (Souza et al., 2023; Kumar et al., 2024). The application of antibodies aimed at neutralizing TNF-α in IBD patients has induced satisfactory clinical responses in up to 60% of patients, leading to disease remission (Souza et al., 2023). Herein, the level of IL-1β, IL-6 and TNF-α was decreased after KF treatment, implying that KF can inhibit the inflammatory response *in vivo*. α-SMA is a marker protein of smooth muscle cells, whose upregulation is associated with more severe tissue fibrosis (Mancarella et al., 2022; Vaes et al., 2024). The expression of α-SMA was upregulated in the model group *in vivo*, but was decreased in the KF treatment group, hinting that KF may inhibit the activation of fibroblasts and thereby alleviate intestinal fibrosis. Moreover, increased permeability is related to the pathogenesis of intestinal diseases. We also found that KF treatment significantly increased the TER of isolated intestinal tissue. *In vitro* and *in vivo* studies have shown that the downregulation of the scaffold protein ZO-1, encoded by the ZO1 tight junction protein (TJP1) gene, correlates with increased tight junction permeability (Ibrahim et al., 2020; Kuo et al., 2021). In the model group, the expression of ZO-1 was downregulated and the intestinal mucosal barrier function was impaired. However, KF treatment could improve the intestinal mucosal barrier function possibly by regulating ZO-1 expression.

In cell experiments, KF intervention reversed the suppressing effect of TNF-α on ZO-1 and HMOX1 proteins in intestinal epithelial cells HT29, while inhibiting the upregulation of TGF-β1 and HO-1. Intestinal infection is an important contributor to the development of IBD. Studies have confirmed that HMOX1 promotes bacterial clearance and reduces intestinal inflammation (Sebastián et al., 2018; Zhan et al., 2018). HMOX1 plays a role in bacterial infections not only by regulating immune responses, but also through a direct interaction with pathogens (Sebastián et al., 2018). In chronic IBD, the persistent high expression of TGF-β1 accelerates intestinal fibrosis progression and seriously affects the structure and function of the intestine (Wang et al., 2022). Our research indicated that KF regulated the function of intestinal epithelial cells and alleviated inflammatory responses *in vitro*. The HT29 cell culture medium containing TNF-α was co-cultured with fibroblasts CCD-18Co. It was found that TNF-α activated CCD-18Co cells, which was reversed by KF, indicating that KF could attenuate the activating effect of intestinal epithelial cell-derived factors on fibroblasts.

Further research has found that HMOX1 was instrumental in KF-mediated regulation of crosstalk between intestinal epithelial cells and fibroblasts. By manipulating HMOX1 expression in the HT29 cell line, we demonstrated that HMOX1 silencing reversed the inhibitory effect of KF on fibroblast activation. This indicated that KF may regulate the crosstalk between intestinal epithelial cells and fibroblasts via regulating the expression of HMOX1, thereby intervening in IBD. In addition, HGF promotes the repair of intestinal epithelial cells, regulates immune responses and inhibits intestinal fibrosis in enteritis (Usunier et al., 2021). KF treatment in fibroblast CCD-18Co reversed TNF-α-induced suppression of fibroblast-mediated HT-29 cell activity, and regulated the level of HGF. Thus, KF can mediate the function of fibroblasts and affect their interaction with intestinal epithelial cells, thereby exerting a therapeutic effect on IBD.

However, although this study discovered the regulatory effects of KF on multiple signaling pathways, the interrelationships among these signaling pathways and their synergistic effects in IBD have not been fully clarified yet. Furthermore, the metabolic process of KF in the body and the influence of its metabolites on IBD have not been deeply studied either, which may affect the comprehensive evaluation of the therapeutic effect of KF. It is worth noting that the exclusive use of female mice in this study may ignore potential sex-based differences in colitis pathogenesis, and this gender bias is an unavoidable limitation of our experimental design. In addition, the barrier-related conclusions were primarily derived from HT29 cells, whose physiological characteristics may differ from those of normal intestinal epithelial cells, potentially limiting the generalizability of the finding that KF affects the intestinal barrier. Finally, in future studies, we will incorporate an *in vivo* HMOX1 inhibition/knockdown strategy to verify whether the efficacy of KF is weakened, thereby completing the logical chain of evidence.

In conclusion, this study has revealed the therapeutic effect and mechanism of KF in IBD. By regulating the crosstalk between intestinal epithelial cells and fibroblasts, it inhibits inflammatory responses, alleviates intestinal fibrosis, and improves intestinal mucosal barrier function, thereby providing new targets and theoretical basis for the treatment of IBD. In the future, we will investigate the Nrf2 pathway and other inflammatory pathways to further elucidate the mechanism of action of kaempferol, thereby laying a more solid foundation for its clinical application.

## Data Availability

The raw data supporting the conclusions of this article will be made available by the authors, without undue reservation.
